# Self-Healing Supramolecular Hydrogels Based on Reversible Physical Interactions

**DOI:** 10.3390/gels2020016

**Published:** 2016-04-08

**Authors:** Satu Strandman, X.X. Zhu

**Affiliations:** Département de Chimie, Université de Montréal, C.P. 6128, Succursale Centre-ville, Montreal, QC H3C 3J7, Canada; satu.strandman@umontreal.ca

**Keywords:** hydrogels, self-healing, supramolecular materials, non-covalent interactions, dynamic cross-links, physical gels, transient networks, mechanical failure and recovery, self-assembly, host-guest chemistry

## Abstract

Dynamic and reversible polymer networks capable of self-healing, *i.e.*, restoring their mechanical properties after deformation and failure, are gaining increasing research interest, as there is a continuous need towards extending the lifetime and improving the safety and performance of materials particularly in biomedical applications. Hydrogels are versatile materials that may allow self-healing through a variety of covalent and non-covalent bonding strategies. The structural recovery of physical gels has long been a topic of interest in soft materials physics and various supramolecular interactions can induce this kind of recovery. This review highlights the non-covalent strategies of building self-repairing hydrogels and the characterization of their mechanical properties. Potential applications and future prospects of these materials are also discussed.

## 1. Introduction

Hydrogels are soft solids or solid-like materials that immobilize a large amount of water in a three-dimensional (3D) network held together by covalent bonds, non-covalent or topological interactions [1,2,3,4]. These materials are appealing to various applications especially in biology and medicine owing to their compositional and structural versatility that allows introducing responsiveness to external stimuli and adapting them to biological interfaces, high water content and physical properties that are similar to soft tissues or at nanoscale, similar to extracellular matrix [5,6,7,8,9,10,11,12,13]. Some of the applications of hydrogels in (bio)medical fields include sensors [14,15,16,17], actuators [18,19], device coatings [20,21,22], wound dressings and adhesives [23,24,25], liquid-absorbing hygiene products [26], delivery vehicles for active compounds or cells [27,28,29,30], soft contact lenses [31,32,33], and matrices and implants in tissue engineering and regenerative medicine [34,35,36,37].

**Figure 1:**
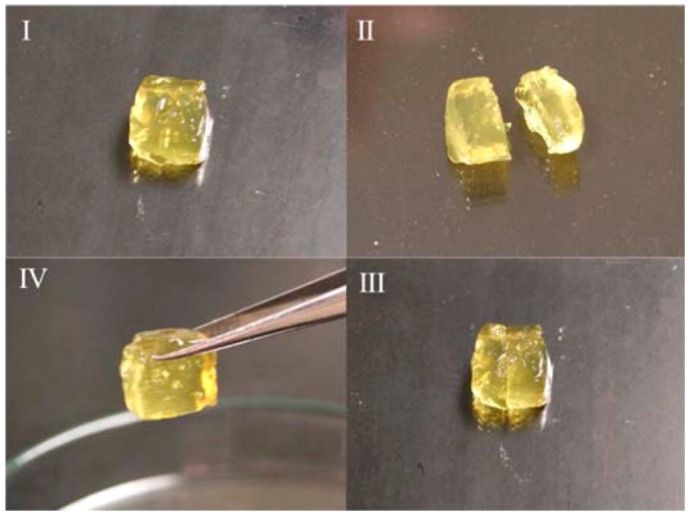
Visual evidence of self-healing of a supramolecular hydrogel through non-covalent host-guest interactions between β-cyclodextrin- and bile acid-bearing polymers: (**I**) initial hydrogel sample; (**II**) sliced sample; (**III**) sliced sample rejoined for healing; and (**IV**) self-healed sample. Reprinted from [63] with permission. Copyright 2016 American Chemical Society.

There is a continuous need towards extending the lifetime, improving the safety, and enhancing the performance of both soft and hard materials. Mechanical deformation may lead to micro- or macroscale cracks, leading to gradual deterioration or sudden loss of mechanical properties [38,39]. One strategy to improve the performance and extend the lifetime of gels is to introduce self-healing ability, that is, a capacity to restore the initial properties after material failure, using dynamic and reversible linkages [39,40,41,42,43,44,45]. A visual demonstration of the self-healing of a hydrogel is shown in Figure 1. The dynamic linkages can be based on reversible covalent chemical bonds, formed for example by Diels-Alder reaction [46,47,48,49,50], disulfide [51,52,53], imine [54,55], oxime [56], or acylhydrazone [50,52] formation, photocrosslinking [57], radical reactions [58,59], or phenylboronate complexation [60,61,62], and these have been highlighted in several reviews. Dynamic linkages can also be based on supramolecular non-covalent (physical) interactions, such as crystallization, hydrogen bonds, host-guest, hydrophobic or polymer-nanocomposite interactions, or multiple combined interactions. Some of these non-covalent networks can be built from pre-formed colloidal systems, such as liposomes or mixed micelles. 

Physical transient gels are considered as a model system in soft materials physics with their well-defined structural properties, ease of preparation, and relatively simple linear rheological behavior, although their non-linear rheological response can be highly complex [38]. Recent reviews have focused on hard and/or chemically crosslinked self-healing materials [41,43,45,48,64] or on certain classes of supramolecular polymers, such as hydrogen-bonded or metal-ligand-coordinated systems [40,42,44,65,66,67]. Wang and Heilshorn [68] highlighted the potential of adaptable hydrogels in cell encapsulation. As non-covalently crosslinked gels often show rapid structure recovery, they are gaining increasing interest in injectable and extrudable systems used in therapeutic delivery or additive manufacturing (3D printing). These applications require the knowledge of the precise recovery rate and mechanical properties of the gels. For example, the tailored elasticity of the extracellular matrix-mimicking hydrogels as bio-inks is crucial for the cell differentiation and survival, while injectable gels should have sufficient strength after structure recovery to hold them in place. These reviews have emphasized the chemistry for the making of the physical gels rather than their resulting physical properties. The self-healing phenomenon is mostly based on visual demonstrations. Further understanding of the relation of the design, structure, and properties is in the call for new developments in this area. We attempt to fill this gap by presenting the preparation strategies in relation to the mechanical properties of the self-healing gels through examples along with a review of the methods used for mechanical testing of self-healing characteristics. We will then discuss the potential applications where gel strength and fast recovery play an important role and present the future prospects of reversible physical gels.

## 2. Characterization of the Self-Healing Behavior

The term “self-healing” has been used to describe various structure recovery processes of hydrogels, where the gels revert to their original state and recover the mechanical strength completely or partially after the deformation or fracture. The gelation and structure recovery can take place via different interactions and self-assembling processes. The viscoelastic properties of physical networks are determined by the number and lifetime of crosslinks, and these further affect the kinetics of the self-repair [69,70]. In addition to macroscopic healing, transient chain association causes temporal evolution of microscopic topology, leading to changes in mechanical properties [71]. Therefore, both macroscopic rheology and microscopic fluorescence results have been used as a basis for theoretical models that aim at quantitative prediction of chain association dynamics in physical networks [69,70,71].

**Figure 2:**
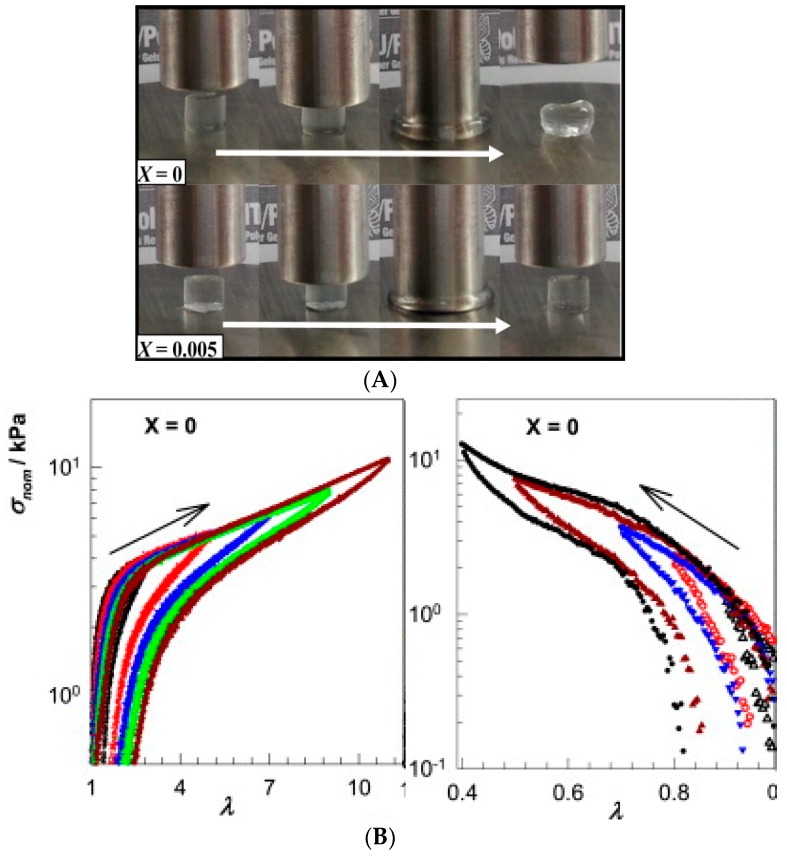
(**A**) Photographs of the physical (molar ratio of chemical crosslinker *vs.* monomer *X* = 0) and hybrid gels with combined chemical and physical crosslinking (*X* = 0.005) during the compression tests. The physical gel remains deformed after removal of the stress (**top-right** image); (**B**) Nominal stress σ_nom_ *vs.* deformation ratio λ curves from cyclic elongation (**left**) and compression tests (**right**) for a physically crosslinked gel (*X* = 0). Reprinted from [72] with permission, Copyright 2016 Elsevier.

Common experimental methods to characterize the self-healing behavior of hydrogels include:

- *Visual observation.* Freshly cut or damaged pieces of a hydrogel are brought in contact and their rejoining to form a uniform gel is followed both visually and using a simple qualitative mechanical deformation, that is, stretching or bending the sample. Examples are shown in Figure 1 and in several figures below. Visual observation can be accompanied by microscopic imaging techniques (confocal, scanning electron microscopy SEM) to reveal the micro- or nanoscale structure recovery.
- *Oscillatory rheology: Step strain measurement.* After determining the yield stress/strain and the recovery time of the gel sample, the gel will be exposed to strain that alternates periodically between low (structure conservation or recovery) and high values (structure breaking) at constant oscillation frequency. This test allows for the quantitative determination of equilibrium moduli and the extent of structure recovery (recovery rate). Several examples of step strain tests will be shown in Section 3.
- *Cyclic compression/tensile testing*. Self-healing of hydrogels can be tested by cyclic compression or tensile tests, each cycle followed by a recovery period. The changes in stress/strain curves and fracture point or in initial compressive modulus give information on the recovery of ruptured crosslinks. Tensile tests can be used to quantify the self-healing efficiency after joining the fractured surfaces of a ruptured hydrogel by comparing the elongation at break for an intact and re-joint hydrogel. Figure 2 shows an example of a compression test of a physically crosslinked hydrogel and a hybrid gel with combined chemical and physical crosslinking, as well as the compression and elongation curves for a physically crosslinked gel (no chemical crosslinker). Here, the nominal stress σ_nom_ is the force per cross-sectional area of the un-deformed gel specimen and the strain is represented by λ, the deformation ratio (deformed length/initial length). The hysteresis in the compression and elongation curves indicates the decreased number of crosslinks and thus, reduced structure recovery over the deformation cycles [72].

One of the challenges of physically crosslinked gels is optimizing the strength and self-healing properties, as the rigidity of gels limits the chain diffusion to the damaged site. Despite the versatility of supramolecular hydrogels, they suffer from relatively low storage moduli (G′ in Pa–kPa range) and so far, combining them with reversible or irreversible covalent networks or harder domains has provided the best mechanical strength. For example, a single component PVA hydrogel with H-bonded crystalline domains could reach a fracture stress of ~280 kPa initially and ~200 kPa after 48 h healing (72% of original strength) [73], and nanocomposite gels of hydrophobically modified polyacrylamide and graphene oxide (GO) could achieve tensile stress of 243 kPa, but the recovery rate was low, 44%. For comparison, the same gel without the reinforcing GO showed a tensile strength of 52 kPa [74].

## 3. Preparation Strategies

### 3.1. Hydrophobic Interactions

Hydrophobicity gives rise to unusual properties of aqueous solutions of nonpolar compounds and plays a key role in a variety of chemical and biophysical phenomena, such as protein folding or self-assembly of amphiphiles into micelles and membranes [75]. Hydrophobic interactions are different from other noncovalent interactions as they do not depend on direct intermolecular attraction between interacting species but are rather driven by the tendency of water molecules to retain their H-bonded network intact around a nonpolar solute, altered by increased temperature, the presence of cosolutes, and size and curvature of nonpolar species [76]. Resulting molecular rearrangement can lead to complex colloidal behavior of amphiphilic molecules in aqueous solutions. Polymer-based hydrogels formed through hydrophobic interactions can be made by introducing hydrophobic sequences within or in the ends of hydrophilic polymer chains [77]. The transient network formation through interchain interactions depends on polymer concentration, fraction of hydrophobic moieties, and polymer architecture [77,78,79]. Such non-covalent hydrogels may exhibit self-healing capacity owing to the dynamic and reversible nature of the junctions [80].

Self-healing associating networks of hydrophobically modified water-soluble polymers have been made by micellar copolymerization of (1) hydrophilic comonomers, such as acrylic acid (AAc), acrylamide (AAm) or *N*-alkylacrylamides (*N*,*N*-dimethylacrylamide, *N*-isopropylacrylamide); with (2) large hydrophobic monomers, such as stearyl methacrylate (C18) [80,81,82,83,84], dococyl acrylate (C22) [81]; octylphenyl polyethoxyether acrylate [85] in the presence of (3) a surfactant (sodium dodecyl sulfate SDS, cetyltrimethyl ammonium bromide CTAB) and depending on the hydrophobe; (4) salt (NaCl, NaBr) or a cosurfactant. Micellar radical copolymerization in aqueous solution is a common technique for the synthesis of associative copolymers. However, unlike smaller hydrophobic *N*-alkylacrylamides or *N*-alkyl(meth)acrylates (C4–C12), long-chain alkyl(meth)acrylates have very low water-solubility. The addition of sufficient amount of salt or cosurfactant induces the growth and/or morphological transition of surfactant micelles. Larger micelles can then solubilize a high amount of hydrophobes, whereupon they can grow further (catanionic CTAB-SDS system) or adopt a different morphology (SDS-NaCl system) for thermodynamic feasibility [81,82]. C18- and C22-acrylamide copolymer gels with a SDS-NaCl system reached final elastic modulus G′ of around 1 kPa at 1 Hz and tan δ of 0.5–0.9, monitored with a rotational rheometer during the polymerization.

**Figure 3:**
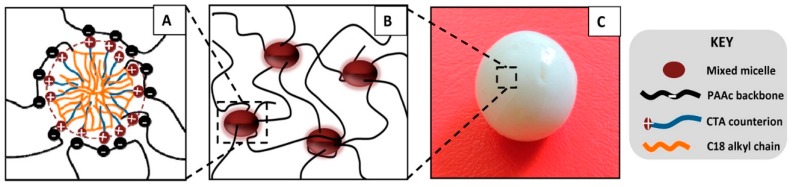
(**A**,**B**) Cartoon showing the cross-link in self-healing hydrophobically modified poly(acrylic acid) (PAAc) hydrogels; (**C**) Image of a PAAc hydrogel sample in the form of a sphere in equilibrium with water. The mixed micelles consist of stearyl methacrylate (C18) and CTAB in aqueous NaBr solution. Reprinted with permission from [80]. Copyright 2016 American Chemical Society.

Okay group found that after the copolymerization, the hydrogels became mechanically stronger upon swelling and extraction of surfactant micelles, as the hydrophobic interactions became enhanced without surfactant [81]. The tensile strength of a C18-acrylamide copolymer gel increased from 12 ± 1 kPa to 78 ± 6 kPa after the removal of SDS micelles by equilibrium swelling in water, but at the same time the elongation at break decreased from 2200% ± 350% to 650% ± 80%, indicating increased stiffness of the gel. At the same time, the gels lost their ability to self-heal because of longer lifetimes of hydrophobic associations. The self-healing property was tested by a tensile strength test on a series of hydrogel samples before and after joining the fractured surfaces of ruptured gels. The results indicate that the self-healing capacity of hydrophobically modified PAAm gels requires the presence of hydrophobe-solubilizing surfactant micelles [81] and possibly a charged comonomer for electrostatic trapping of oppositely charged micelles, such as AAc for trapping mixed micelles of C18 and CTAB (Figure 3) [80,84]. The self-healing efficiency increases with decreasing lifetime of dynamic crosslinks due to favorable chain diffusion across the fractured surfaces. Therefore, improving the gel strength by enchancing the hydrophobic interactions or even combining physical and covalent crosslinking (hybrid gels) [72], takes a toll on the self-healing capacity and balancing these two characteristics is important in optimizing the gel performance.

**Figure 4:**
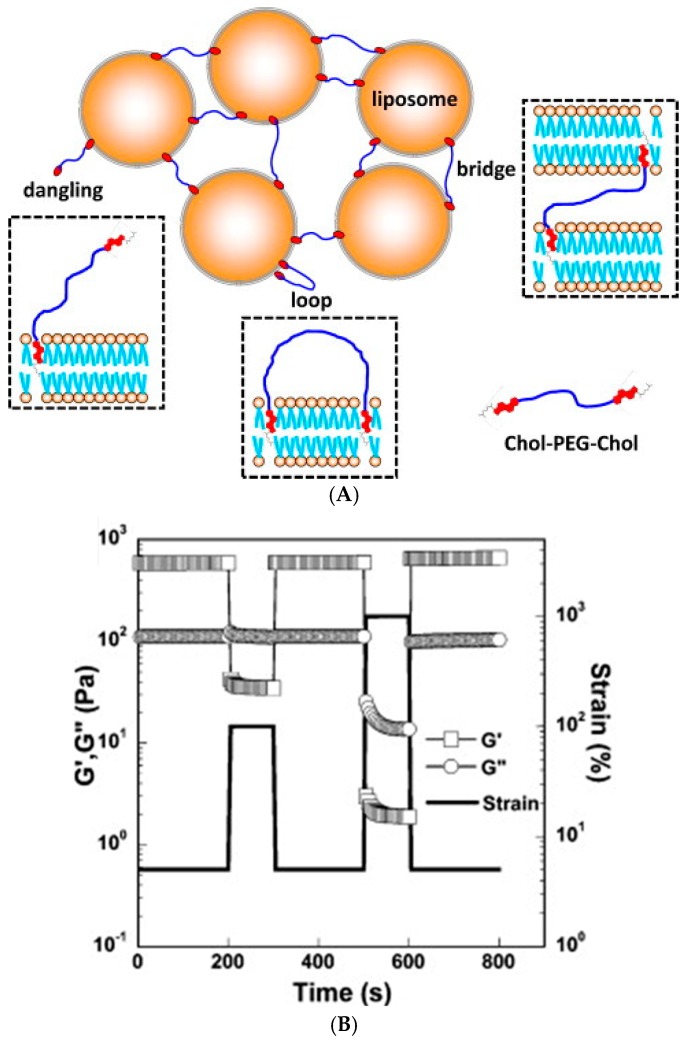
(**A**) Schematic illustration of liposome gel. Cholesterol end groups of Chol-PEG-Chol were embedded in the liposome bilayers, forming bridge, loop or dangling; (**B**) Evolution of storage G′ (□) and loss modulus G″ (○) of liposome gel with time following two successive pulses of high deformation (solid line). Reprinted with permission from [87]. Copyright 2016 Elsevier.

Another method for preparing a hydrophobically associated self-healing hydrogel is introducing reversible network junctions through liposomes that can anchor the hydrophobic moieties of (co)polymers into their bilayer. As liposomes have found clinical applications as drug carriers [86], their delivery to a specific site in the body could be achieved via injectable, rapidly self-healing hydrogels. Mixing a telechelic cholesterol (Chol) end group-bearing poly(ethylene glycol) (PEG) with dimethyldioctadecylammonium bromide (DODAB) liposomes resulted in an elastic self-healing gel where Chol-PEG-Chol acted as a dynamic crosslinker between the liposomes (Figure 4) [87]. High liposome concentration and low temperature favored the network formation through higher probability of bridging and lower mobility of Chol groups into and out of liposome bilayers, respectively. The plateau modulus G_0_ at high oscillatory frequencies in rheological experiments showed a crosslinker concentration (C) dependence of G_0_~C^4.3^ (*vs.* ~C^2.25^ of Cates model for living polymers and flexible wormlike micelles), which suggests that the interactions between the liposome and crosslinker are more complex than those in a simple polymer network. The highest Chol-PEG-Chol concentration (6% *w*/*v*) gave *G*_0_ of 5.5 kPa. The gels showed instantaneous self-healing after subjecting them to low stress (yield stress 59 Pa at *C* = 3% *w*/*v*), which is assigned to the re-insertion of Chol groups into liposomes after stress-induced breaking of Chol-PEG-Chol bridges. This gives promise to applications in injectable drug delivery systems and 3D tissue engineering scaffolds [87]. However, the observed body temperature-induced softening of the gel may limit its medical applications and further tuning of the gel strength may be required.

### 3.2. Host-Guest Interactions

Host-guest interactions represent specific non-covalent interactions that are based on selective inclusion complexation between macrocyclic hosts, such as cucurbit[n]urils, cyclodextrins, crown ethers, calix[n]arenes, resorcinarenes, and pillar[n]arenes, and smaller guest molecules [42,88,89,90]. Although the size of the cavity and portal of a host molecule are good predictors of binding, the selectivity of a host towards a guest goes beyond a simple hole-fitting concept, as solvent effects, multiple binding sites or secondary interactions can be involved [90,91]. Self-healing gels exploiting inclusion complexation can be built by (1) mixing host- and guest-equipped polymers or (2) copolymerizing vinyl group-bearing pre-formed host-guest inclusion complexes with comonomers [42,89]. Although all the macrocycles above show potential for building self-healing gels, mainly cyclodextrins and cucurbit[n]urils have so far been used in fabricating hydrogels and will be discussed more in detail below. Crown ether derivatives have been used to fabricate linear and crosslinked supramolecular polymers owing to their affinity towards cationic species [88,92,93], but self-healing has only been reported for organogels formed upon the complexation of polymer-bound cationic guests by a bis(crown ether) [94] or crown-ether-bearing polymers by bis-ammonium compounds [95].

#### 3.2.1. Cyclodextrins

Cyclodextrins (CDs) are popular hosts in supramolecular chemistry owing their availability and selectivity according to the cavity size (γ-CD > β-CD > α-CD), where hydrophobic and van der Waals interactions between the inner surface of CD ring and hydrophobic guests of suitable size are responsible for inclusion complexation [96,97]. Guest molecules can range from polar compounds such as alcohols, acids, amines, and amino acids to less polar compounds such as linear and branched alkyls, cycloalkanes, aromatic molecules, and steroidal compounds or even to polymeric molecules, such as in the case of poly(pseudo)rotaxanes [98].

**Figure 5:**
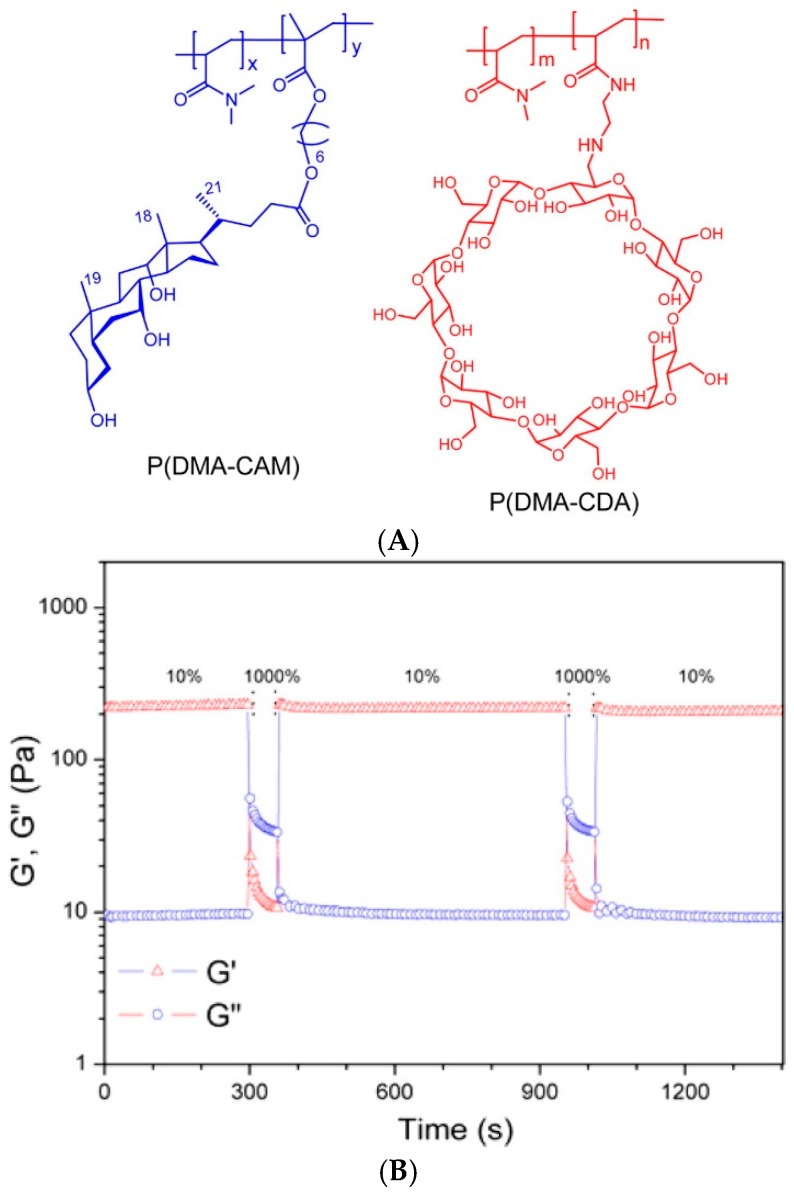
(**A**) Structures of cholic acid- (**left**) and β-CD-bearing (**right**) poly(*N*,*N*-dimethylacrylamides), P(DMA-CAM) and P(DMA-CDA), respectively; (**B**) G′ (squares) and G″ (circles) values of the P(DMA-CAM-2%)/P(DMA-CDA) hydrogel (8.3 wt%) in continuous step strain measurements (25 °C). Large strain (1000%) inverted the values G′ and G″ to give the sol state. G′ was recovered under a small strain (10%) within 30 s. Reprinted with permission from [63]. Copyright 2016 American Chemical Society.

Following the first strategy of mixing two functionalized polymers, self-healing gels have been prepared from CD-bearing polymers, such as poly(meth)acrylates, poly(*N*-alkyl)acrylamides, poly(ethylene glycol) or poly(ethylene imine), and guests such as ferrocene [99,100,101,102], imidazole [103], bromonaphthalene [104], and azobenzene [104]. Even larger guests can be bound, such as bile acids, which are physiologically important steroidal compounds and thus, ideal building blocks for polymeric biomaterials [105,106,107,108]. They can form inclusion complexes with β-CD and the complexation can be inversed upon the addition of competing guest, potassium 1-adamantylcarboxylate [109]. Host-guest complexation between poly(*N*,*N*-dimethylacrylamide) chains bearing either β-CD (8 mol%) or cholic acid moieties (2 mol%) yielded self-healing hydrogels, which showed highest storage modulus G′ at 1:1 host guest ratio and high polymer concentrations (12.5 wt%). The gel elasticity was recovered rapidly (in 30 s) after the shear-induced rupture of supramolecular crosslinks (Figure 5). The plateau modulus G_0_ of 1:1 gels was ~10–1000 Pa at 6.5–12.5 wt% [63]. Another steroidal molecule, cholesterol (Chol), has been used as a guest in self-healing gels based on the inclusion complexation between poly(l-glutamic acid) (PLGA)-bound β-CD (48 mol%) and PLGA-*b*-PEG-*b*-PLGA triblock copolymer furnished with pendent Chol groups (20 mol%) [110]. The healing time of these gels was 60 s, determined by oscillatory step strain experiments, and high compressive modulus (46 kPa) was obtained with a high-molar-mass linker with long PLGA blocks (~30 kDa). The gels showed good biocompatibility and slow degradation of up to 72 days *in vitro* at 37 °C [110]. 

**Figure 6:**
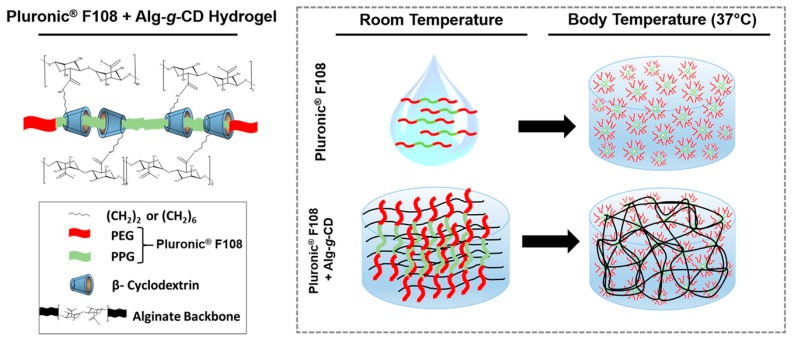
Physical cross-linking between Alg-*g*-CD macromolecules and Pluronic^®^ F108 (**left**) and the thermo-response of the hydrogel network (**right**). Pluronic^®^ F108 forms micelles and self-cross-links at body temperature. β-CD (host) conjugated onto the alginate backbone (Alg-*g*-CD) formed a physically cross-linked supramolecular inclusion complex with the guest, the PPG block (green) of Pluronic^®^ F108. Reprinted with permission from [111]. Copyright 2016 American Chemical Society.

Self-healing can also be achieved through reversible polypseudorotaxane formation, which involves binding of a polymer chain inside several polymer-bound macrocyclic hosts. Complexation between β-CD-grafted alginate (Alg-*g*-CD) and Pluronic^®^ 108 yielded self-healing biocompatible and degradable hydrogels (Figure 6), where multiple cyclodextrin hosts bound to the middle block of Pluronic PEG-*b*-PPG-*b*-PEG triblock copolymer (PPG = poly(propylene glycol)) [111]. Rapid recovery from shear (in 10 s) was observed and the gels remained stable over long periods of time (up to 5 days). As Pluronic^®^ 108 is thermosensitive, forming micelles at body temperature, the association of the β-CD-bound block copolymer upon increasing temperature led to significant stiffening of gels and the strongest gels showed elastic shear modulus *G*′ of 10–12 kPa at *f* = 10 Hz. The gels exhibited gradual release of a globular protein of 66.5 kDa (bovine serum albumin, BSA) *in vitro* and thus, applications as injectable delivery vehicles were proposed [111].

**Figure 7:**
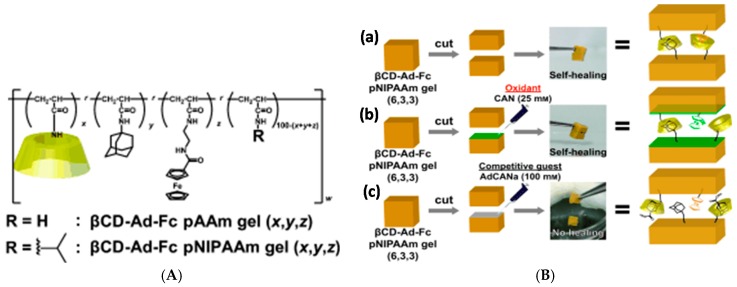
(**A**) Structures of copolymers bearing β-CD, adamantane (Ad) and ferrocene (Fc) in the same chain; *x*, *y* and *z* indicate mol% of respective units in the chain; (**B**) Self-healing properties of a poly(*N*-isopropylacrylamide) copolymer (**a**) with Fc in reduced form; (**b**) Fc in oxidized form; and (**c**) in the presence of competitive guest sodium adamantanecarboxylate (AdCANa). Reproduced with permission from [113]. Copyright 2016 John Wiley and Sons.

The host and guest moieties can also coexist in the same polymer. Harada group prepared a series of tough and flexible self-healing gels from a variety of polyacrylamides and polyacrylates, where β-CD and adamantane were side groups of the same polymer chain, leading to interpolymer interactions and thus crosslinking [112]. High host and guest content in the same chain led to poorer mechanical properties through the heterogeneity of the resulting network. The polymers showed potential as self-healing coatings in the dry state, where healing through supramolecular interactions could be induced upon exposing the damaged area to water that acts as a plasticizer and increases the mobility of the polymer chains. The synthetic approach was taken even further in redox-sensitive self-healing gels based on polyacrylamides furnished with the abovementioned β-CD and Ad units as well as a ferrocene side group, which can be bound to β-CD in its reduced state but expelled in oxidized form (Figure 7) [113]. The strongest gels based on β-CD-Ad-Fc-PNIPAAm achieved Young’s modulus E of up to 20 kPa, obtained by tensile testing. Self-healing occurred in both oxidized and native states although the healing efficiency was lower for the oxidized gel. Here, the self-healing took at least 2 h and 70% recovery ratio was achieved for native gel in >70 h. Oxidization also led to gel swelling due to reduced crosslinking density. Interestingly, the gels showed also a shape memory effect based on the release of Fc upon oxidation and rebinding to a new β-CD site upon reduction [113].

**Figure 8:**
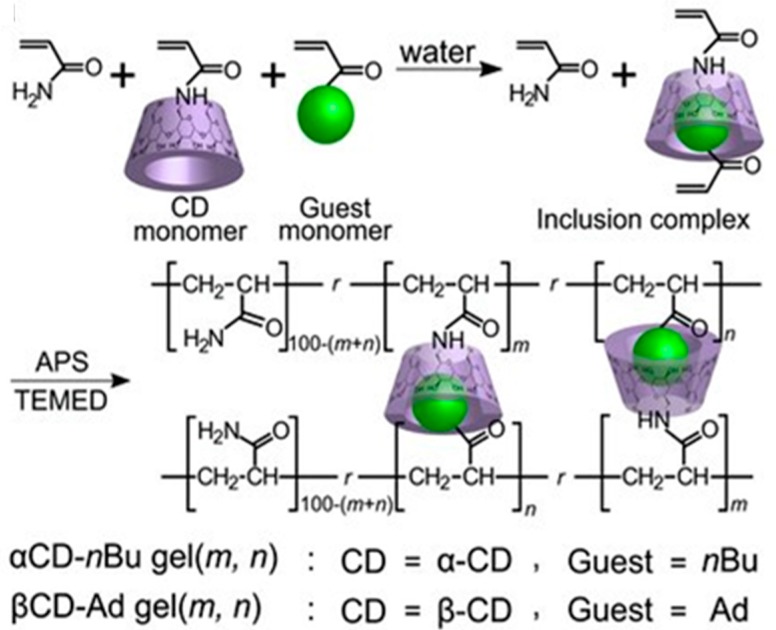
Preparation of host-guest supramolecular αCD-nBu gels (*m*, *n*) and βCD-Ad gels (*m*, *n*); *m* and *n* denote the mol% of the host unit and guest unit, respectively. Reprinted with permission from [114]. Copyright 2016 John Wiley and Sons.

Figure 8 presents an example of the second type of preparation strategy for self-healing supramolecular gels, where host-guest complexes are formed prior to the copolymerization of host (α-CD or β-CD) and guest (adamantane, Ad or n-butyl, n-Bu) molecules that both bear polymerizable vinyl groups [114].The aim was to improve the self-healing capacity upon preorganization of interacting domains. Greater degree of self-repair was obtained with Ad guest (99% after 24 h, bound to β-CD) than with n-Bu (74% after 24 h, bound to α-CD) and the elastic modulus G′ of β-CD-Ad gel was 100–1000 times higher than that of α-CD-nBu gel (680 kPa *vs.* 0.33 kPa). Self-healing of the former gel could be prevented upon adding a competitive guest, sodium adamantanecarboxylate (AdCANa), or free host, β-CD [114]. 

#### 3.2.2. Cucurbit[n]urils

Macrocycles composed on glycouril units, cucurbit[n]urils (CB[n], where *n* = 5–8, 10; *n* = 6 most abundant), can form binary 1:1 or ternary 1:1:1 host-guest complexes with a variety of guest molecules. A comprehensive review of guests of CB[n] was recently published by Scherman *et al.* [115]. The cavity of CB[8] is large enough to accommodate two guests, and ternary complexes can be formed upon simultaneous binding of an electron-rich and an electron-deficient guests, such as naphthyl and methylviologen derivatives, respectively [116,117]. Because the functionalization of cucurbit[n]urils can be challenging [118,119], inclusion complexation-induced crosslinking and gelation is rather achieved upon ternary binding of polymer-bound guests by free CB[n] [120,121,122].

**Figure 9:**
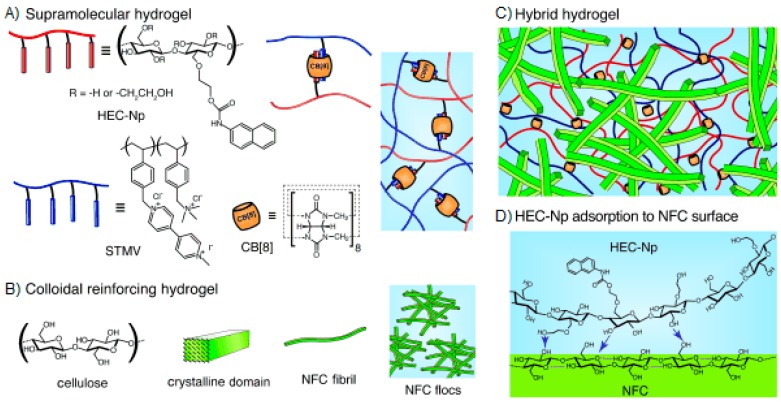
(**A**) Supramolecular hydrogel consisting of HEC-Np, STMV, and the CB[8] host motif capable of binding the first guest naphthyl and the second guest viologen highly dynamically; (**B**) Colloidal reinforcing nanofibrillated cellulose, also showing the denser and less dense network regimes; (**C**) Interpenetrating hybrid hydrogel consisting of the molecular-level supramolecular and colloidal-level NFC hydrogel; and (**D**) Surface adsorption of HEC-Np onto the NFC surface. Possible hydrogen bonding is schematically shown. Reprinted with permission from [122]. Copyright 2016 John Wiley and Sons.

Self-healing hydrogels were prepared from naphthyl-functionalized hydroxyethyl cellulose (HEC-Np) and a viologen-functionalized poly(vinyl alcohol) (PVA-MV) [121]. The latter gels were reinforced by adding colloidal nanofibrillated cellulose (NFC) hydrogel to a fixed composition of supramolecular HEC-Np/PVA-MV/CB[8] hydrogel. Figure 9 depicts the resulting interpenetrating network (IPN). Supramolecular hydrogel alone showed rapid and complete self-healing after repeated shear, owing to rapid association kinetics of the ternary complex of CB[8] (association constant *k*_a_ ~10^8^ M^−1^·s^−1^) [121]. The strongest hybrid hydrogel has elastic modulus G′ of 2 kPa at 0.1% strain and it was stronger (up to 50-fold increase in G′), had higher yield strain (up to >4-fold enhancement) and showed improved and faster structure recovery than NFC gel alone. Supramolecular HEC-Np/PVA-MV/CB[8] hydrogel bridged the denser floc-like domains of colloidal NFC gel and mediated the healing of NFC network [122]. The recent research on ternary CB[n] complexes [115,123,124,125] gives promise to further developments in self-healing host-guest materials.

### 3.3. Hydrogen Bonding

**Figure 10:**
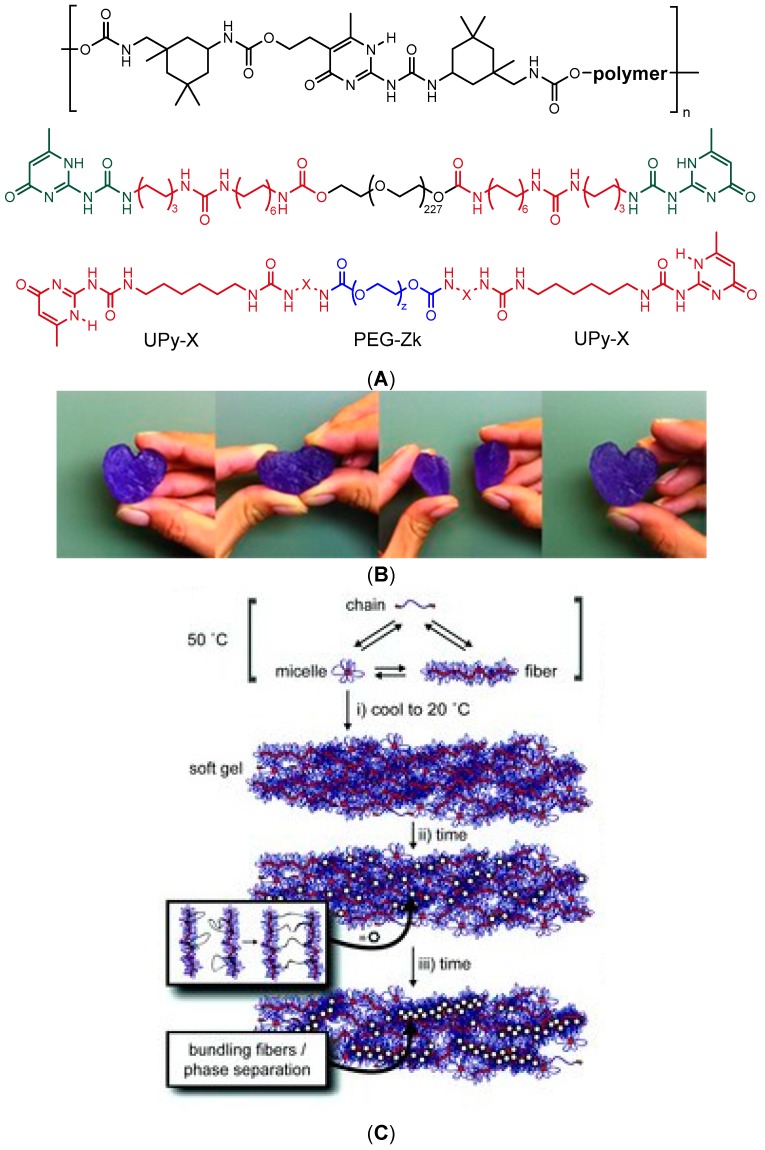
(**A**) Examples of the structures of PEG-based H-bonding polymers that contain UPy units as part of main chain [128] or as end groups [129,130]; (**B**) Visual demonstration of the self-healing of a 15 wt% hydrogel of the main-chain-functionalized polymer of (a). Purple dye was added for demonstration purposes [128]; (**C**) At high temperatures (above 50 °C) and in dilute solution, the hydrogelators are present as three species: single chain, spheric micelle, and fiber: (**i**) Upon cooling, or increase in concentration, a soft hydrogel is formed; (**ii**) After a time span of approximately 16–24 h, the strength of the gel is increased due to the formation of supramolecular cross-links; and (**iii**) The fibers may bundle and phase separate forming ordered domains in the hydrogel network. Reprinted with permissions from [128,129]. Copyright 2016 John Wiley and Sons.

Dynamic supramolecular polymers are typically synthesized by introducing complementary H-bonding donor and acceptor motifs into the building blocks [65,66,126], which is also an elegant strategy for building reversible networks with self-healing capacity. In aqueous medium, the H-bonding occurs in competition with water molecules, whose contribution can be diminished by using multiple H-bonding motifs with high dimerization affinity, such as 2-ureido-4[1*H*]pyrimidinone (UPy) units. The dimerization can be further enhanced by shielding the motifs from water with hydrophobic substituents, such as adamantyl (Ad) or alkyl groups [127]. Such motifs have been used to induce reversible physical network formation of a copolymer of *N*,*N*′-dimethylacrylamide (DMA) and a methacroyl monomer bearing a Ad-functionalized UPy unit [127] or a PEG chain bearing UPy moieties shielded from water by apolar isophorone [128] or alkyl spacers [129,130] (Figure 10a,b). The transient network formation of telechelic UPy-bearing polymers arose from the entanglement of supramolecular self-assembled fibrils at elevated concentrations (Figure 10c), which was further dependent on the length of the alkyl spacer and temperature [129]. These hydrogelators showed a reversible transition from viscous liquids to solid-like gels upon cooling, and the gel strength and self-healing properties could be modulated by the addition of short-chain monofunctional UPy-PEGs [130]. The strongest gel showed elastic modulus G′ of 18 kPa at 20 °C at angular frequency of 10 rad/s. Despite the multivalency of interactions, the structure recovery of the gels could take from hours to days, similar to many low-molecular-weight hydrogelators (LMWHs) that also form networks of bundled self-assembled fibers, studied extensively by the groups of Weiss, van Esch, and Zhu, among others [131,132,133,134]. The slow recovery raises a question of whether such systems can be strictly considered as self-healing materials, as rapid healing and fast macromolecular dynamics are often required for potential applications. Nevertheless, the transient network formation of telechelic UPy-alkyl-PEGs was fast enough for the successful encapsulation and injection delivery of an anti-fibrotic growth factor protein BMP7 *in vivo* in the kidney capsule of rats [129].

**Figure 11:**
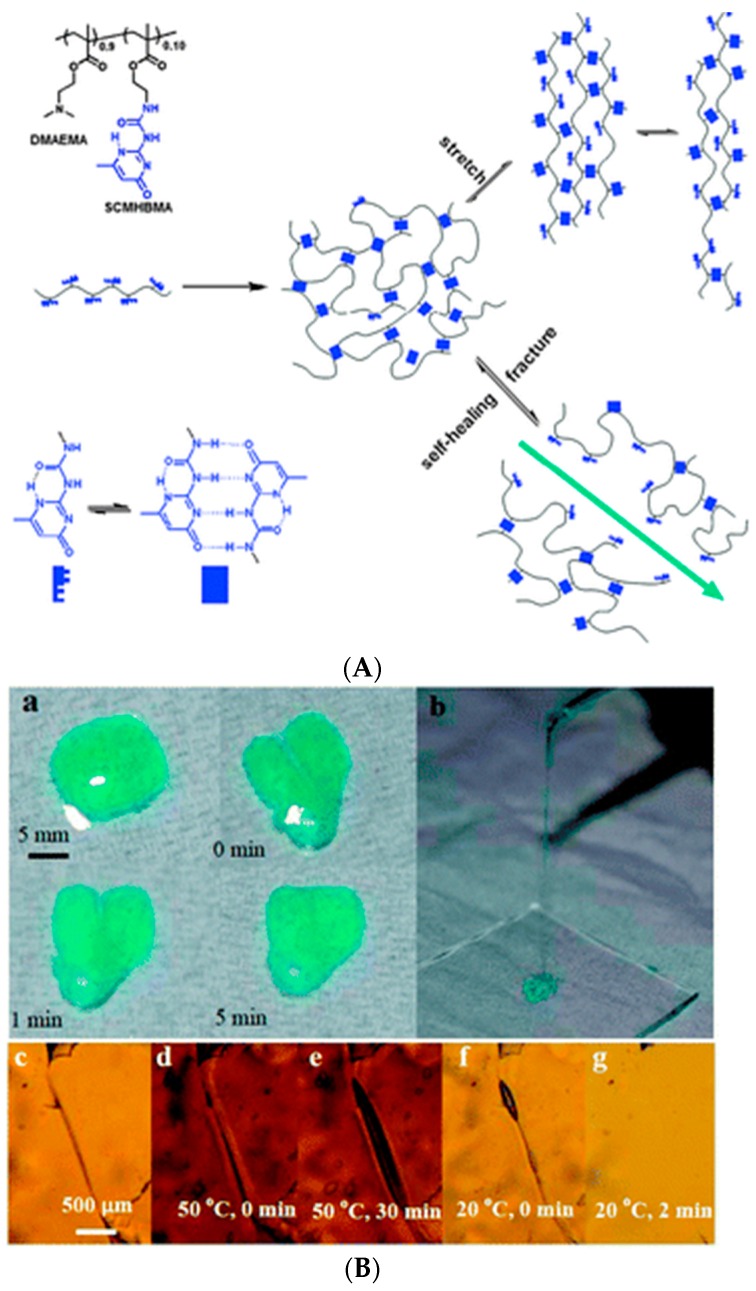
(**A**) Chemical structure of the copolymer containing DMAEMA and SCMHBMA and the schematic model of self-healing and stretching of the hydrogel formed by the copolymer; (**B**) Demonstration of the self-healing (**a**,**c**–**g**) and the stretching (**b**) properties of the DMAEMA-SCMHBMA hydrogel at pH 8. The gel in (**a**,**b**) was colored with methyl blue for better imaging. Optical microscopy images (**c**–**g**) were obtained from a hydrogel film with an incision (**c**) after annealing at 50 °C (**d**,**e**) and subsequent cooling to 20 °C (**f**,**g**). Reprinted with permission from [135]. Copyright 2016 Royal Society of Chemistry.

UPy units can also be introduced as side groups that form interchain crosslinks upon H-bonded dimerization. A copolymer composed of 2-(dimethylamino)ethyl methacrylate (DMAEMA) and a UPy-unit-bearing monomer 2-(3-(6-methyl-4-oxo-1,4-dihydropyrimidin-2-yl)ureido)ethyl methacrylate (SCMHBMA) (Figure 11A) was thermo-responsive with lower critical solution temperature (LCST) of 40 °C at pH 8 [135]. The copolymer yielded viscous solutions at acidic pH where DMAEMA units were protonated, whereas self-healing hydrogels were obtained at neutral to basic pH (pH 7–8) at 20 °C (Figure 11B). Here, the self-healing properties were only observed visually and using polarized optical microscopy (POM). Increasing the temperature above the LCST prevented self-healing, because the collapse of polymer chains restricted their diffusion to the damaged site and H bonds could not be restored. Self-healing was also observed for covalently crosslinked gels with the same composition, as well as for other copolymers of SCMHBMA with hydrophilic or thermo-sensitive comonomers, such as 2-hydroxyethyl methacrylate (HEMA), 2-(2-methoxyethoxy)ethyl methacrylate (MEO2MA), *N*-isopropylacrylamide (NIPAAm), and *N*,*N*′-dimethylacrylamide (DMA) [135]. Later, this monomer was copolymerized into crosslinked polyurethane-PEG-methacrylate (PU-PEGMA) networks to yield highly deformable (elongation at break up to 2000%) self-healing hydrogels, which were capable of recovering up to 87% of their original tensile strength of 382 kPa in 10 min after bringing the cut pieces into contact. The gels also showed high compression strength of up to 4.5 MPa and did not rupture even when exposed to 90% compressive strain [136].

Varghese and coworkers [137] showed that poly(6-acryloyl-6-aminocaproic acid)-based hydrogels (PA6ACA) underwent rapid (<2 s) and repeatable H-bonding-induced self-repair in acidic conditions (pH ≤3) where carboxylic acid groups were protonated. A6ACA monomer contains an alkyl spacer between the acrylamide and –COOH group. The hydrogels healed for 10 s could withstand higher than 2 kPa tensile stresses and the rupture occurred within the bulk region of the gel, not at the welded interface. The self-healing ability depended strongly on the availability of amide bond for H-bonding, that is, the length of the spacer between –COOH and amide group. Based on proof-of-concept testing, the gels are expected to find their applications as acid-resistant sealants, muco-adhesive tissue adhesives and drug delivery vehicles, and soft structures for various devices [137]. Self-healing of these gels could also be induced by divalent metal complexation (Cu^2+^) by A6ACA units [138]. Recently, the copolymers of an amino acid-based monomer, *N*-acryloyl glycinamide (NAGA) and acrylamide (AAm) showed thermoplasticity and temperature-driven H-bonding between the amino acid moieties, which yielded strong self-healing hydrogels (G′ up to 1 MPa) with the healing efficiency of up to 84% [139].

### 3.4. Ionic Interactions

Ionic interactions have long been used to reinforce elastomeric materials, as a relatively small concentration of acid or ionic groups can substantially alter the physical, mechanical, optical, dielectric, and dynamic properties of a polymer [140,141]. Ionomers, *i.e.*, copolymers with typically less than 15 mol% of ionic groups, and polyelectrolytes are widely used to create ionic supramolecular systems and crosslinked networks [141]. For example, self-healing materials based on non-aqueous ionic networks have been made from poly(acrylic acid) (PAA) and a phosphonium ionic liquid linker, where the length of the alkyl substituents and number of phosphonium groups (di- or monofunctional) determined the mechanical properties of the material [142]. Ionic crosslinks can also be used to dissipate energy and induce healing of covalently crosslinked hydrogels. For instance, tough stretchable hydrogels have been synthesized by mixing ionically crosslinked alginate (crosslinking by Ca^2+^ ions) into a photocrosslinked polyacrylamide (PAAm) network. These gels were stronger than individual PAAm or alginate gels and stretchable to nearly 20 times of their length prior to rupture. Of the network strength, 74% was recovered after 1 day of healing at elevated temperature (80 °C) [143].

**Figure 12:**
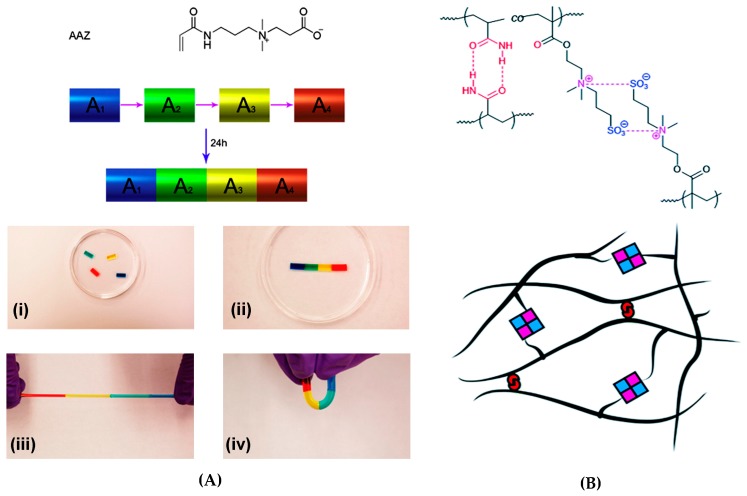
(**A**) Carboxybetaine acrylate (AAZ) monomer and the fusion of its homopolymer hydrogels from separate blocks (A_1_–A_4_, (**i**)) into a uniform gel (**ii**) that can be stretched (**iii**) and bent (**iv**) without breaking. A_1_ (**blue**), A_2_ (**green**), A_3_ (**yellow**) and A_4_ (**red**) have the same composition [144]; (**B**) Reversible hydrogen bonding and electrostatic interactions of a P(AAm-*co*-DMAPS) copolymer, resulting in crosslinked hydrogel formation. Reprinted with permissions from [144,145]. Copyright 2016 Elsevier and Royal Society of Chemistry.

The interacting charges may also exist in the same monomer. Zwitterions are dipolar species, in which the cation and the anion are separate in the same monomer unit and can be completely dissociated, thus maintaining the overall electroneutrality. Zwitterionic polymers have been shown to form reversible self-healing hydrogels through ionic interactions. Examples of such systems are carboxy- or sulfobetaines and their copolymers with neutral comonomers. A carboxybetaine acrylate (AAZ) homopolymer yielded biocompatible rapidly healing ( ≤100 s) hydrogels, where the strongest physically crosslinked gels exhibited elastic modulus G′ of ~800 Pa, tensile strength of around 60 kPa and Young’s modulus E of ~25 Pa. The self-healing of gels (Figure 12a) was induced by the electrostatic attraction (“zwitterionic fusion”) between the quaternary ammonium group and carboxylate group of the same monomer. The self-healing could take place even after a long separation time of the cut fragments both in physically and chemically crosslinked gel samples [144], while most examples of self-healing of gels have been tested on fresh surfaces. Moreover, the fusion of cell-laden pieces of hydrogel did not cause a significant loss in cell viability, which suggests potential applications in tissue engineering and regenerative medicine.

In another carboxybetaine-based system, nanocomposite hydrogels were prepared by the copolymerization of a carboxybetaine methacrylamide CBMAA-3 ((3-methacryloylaminopropyl)- (2-carboxyethyl)dimethylammonium-(carboxybetaine methacrylamide)) and 2-hydroxyethyl methacrylate (HEMA) in the presence of an inorganic clay, Laponite XLG [146]. Laponites are hydrophilic disk-shaped inorganic clay platelets that can be uniformly dispersed in water and act as physical crosslinkers. The presence of Laponite enhanced the mechanical properties of composite hydrogels (Young’s modulus up to 80 kPa, elongation at break up to 1800%) and reduced the water uptake of gels due to higher crosslinking density, while the higher zwitterion (CBMAA-3) content was associated with faster structure recovery [146]. These materials were proposed to act as non-fouling protein-resistant biomaterials. Interestingly, the nanocomposite hydrogels of Laponite with the copolymers of DMA and a sulfobetaine acrylamide (*N*,*N*-dimethyl(acrylamidopropyl) ammonium propane sulfonate) (DMAAPS) demonstrated upper critical solution temperature (UCST) behavior with UCST of ≤9.3 °C when DMA content was ≤10 mol% [147]. The hydrogels showed self-healing behavior above but not below the UCST, similar to the H-bonded LCST network described in Section 3.3 [135], because the diffusion of dangling polymers chains across the damaged region and interactions with clay platelets were disrupted upon the collapse of chains below the UCST. Here, the strongest gel showed elastic modulus G′ of ~1.6 kPa at 0 °C and *f* = 1 Hz, the tensile strength below 5 °C was ~65 kPa and elongation at break was ~1800%. However, only 20% recovery in tensile strength was achieved after 24 h self-healing above the UCST [147].

Combined electrostatic and H-bonding interactions were responsible for the self-healing of a zwitterionic hydrogel based on P(AAm-*co*-DMAPS) polymers, where AAm = acrylamide and DMAPS = 3-dimethyl(methacryloyloxyethyl) ammonium propane sulfonate (Figure 12b) [145]. High healing efficiency (up to 80%) and fast recovery (~35 s) were observed for gels with AAm/DMAPS = 1 and the strongest gel showed elastic modulus G′ of ~5.5 kPa. The suggested applications of these hydrogels are in the field of enhanced oil recovery (EOR) [145].

### 3.5. Polymer-Nanocomposite Interactions

**Figure 13:**
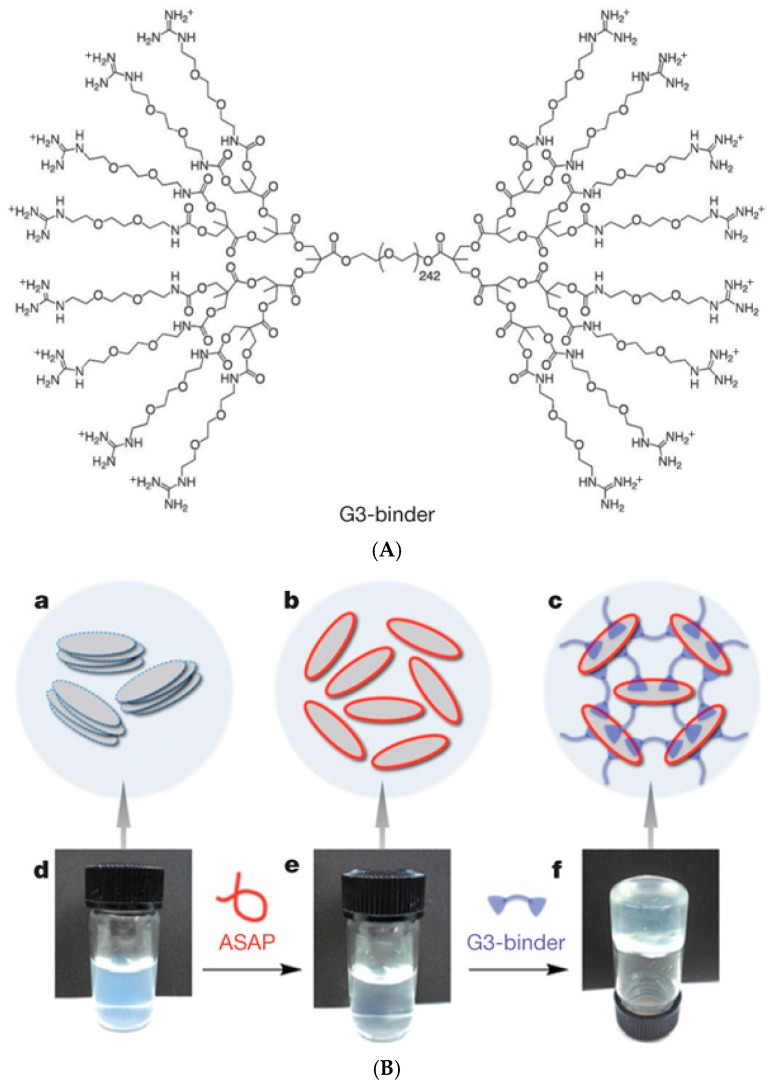
(**A**) Structure of guanidine-functionalized G3-binder; (**B**) Non-covalent preparation of hydrogels. (**a**–**c**) Proposed mechanism for hydrogelation. CNSs, entangled with one another (**a**), are dispersed homogeneously by interaction of their positive-charged edge parts with anionic ASAP (**b**). Upon addition of Gn-binder, exfoliated CNSs are crosslinked to develop a 3D network (**c**). (**d**–**f**) Optical images of an aqueous suspension of CNSs (**d**), an aqueous dispersion of CNSs and ASAP (**e**) and a physical gel upon the addition of G3-binder to the dispersion (**f**). Reprinted with permission from [148]. Copyright 2016 Nature Publishing Group.

Some of the above-mentioned examples have demonstrated the use of inorganic clay nanoplatelets (Laponite) [146,147] to enhance the mechanical properties of supramolecular hydrogels. Aida and coworkers [148] prepared rapidly self-healing hydrogels from aqueous clay nanosheet (CNS) solution with sodium polyacrylate (ASAP) as a dispersant and exfoliator by adding a guanidine-functionalized dendritic macromolecule (G3-binder, Figure 13). Positively charged guanidium groups adhere to anionic CNSs and act as crosslinkers, leading to strong (G′ >5 kPa) free-standing gels which recovered repeatedly from shear-induced rupture in 600 s. The gels could resist brine and moderately acidic and basic conditions (pH 4.0–10.0) and could incorporate and maintain biologically active proteins, such as myoglobin. A myoglobin-loaded gel retained ~71% of the catalytic activity of myoglobin in the oxidation of *o*-phenylenediamine with H_2_O_2_ in phosphate buffer, suggesting potential applications in the transport of biologically active agents [148]. Guanidinium-based (PG_n_) blocks have also been used to build luminescent self-healing hydrogels via physical crosslinking of PG_n_-*b*-PEO_230_-*b*-PG_n_ block copolymers with anionic nanosized inorganic transition-metal oxide clusters, polyoxometalates (POMs). The resulting self-healable hydrogels showed enhanced luminescence properties, elastic modulus G′ of up to 30 kPa and rapid structure recovery in 20 s [149]. In another example of nanoclay composites, the interaction of a cationic PDMAEMA_6_-*b*-PEO_109_-*b*-PDMAEMA_6_ block copolymer with Laponite XLG clay platelets resulted in a self-healing CO_2_-responsive hydrogel, where protonated PDMAEMA blocks were bound to the anionic surface of nanoclay and formed interplatelet bridges. The protonation of PDMAEMA was induced by bubbling CO_2_ through the clay-polymer solution and it could be reversed by N_2_ flow, while the self-healing of the gel arose from the electrostatic interactions. The strongest gel showed elastic modulus G′ of 5 kPa at *f* = 1 Hz [150]. The interaction with nanoclay could also be based on H-bonding, such as in the case of self-healing Laponite nanoclay composites with water-soluble poly(*N*,*N*′-dimethylacrylamide) (PDMA) or thermo-responsive poly(*N*-isopropylacrylamide) (PNIPAAm) [151].

Self-healing hydrogels based on hydrophobic interactions between a hydrophobically modified water-soluble polymer and graphene oxide (GO) sheets were prepared by the copolymerization of stearyl methacrylate and acrylamide in the presence of GO and sodium dodecyl benzene sulfonate (SDBS) [74]. As discussed in Section 3.1, hydrophobically modified copolymers alone can show self-healing properties. Here, the GO sheets acted as physical crosslinking junctions resulting in a dramatic increase in mechanical strength but decreased self-healing efficiency (53% and 66% with 5 wt% GO and without GO, respectively). The strongest gels exhibited tensile strength of 243 kPa with elongation at break of 1700% and elastic modulus of ~65 kPa. The nanocomposite gels could efficiently remove hydrophobic compounds from water, tested by the absorption and release of methylene blue (MB) and congo red (CR) dyes, and could be recycled cyclically for dye absorption without compromising the mechanical properties. Hence, applications in water purification have been suggested [74]. Other self-healing GO nanocomposites have been made for example from chitosan via ionic interactions between cationic chitosan and the anionic surface of GO sheets [152] or poly(6-acryloyl-6-aminocaproic acid) (PA6ACA) in the presence of Ca^2+^ via combined coordination and H-bonding interactions [153].

### 3.6. Other Crosslinking Mechanisms: Crystallization, Transient Protein Interactions

Crystallization of macromolecules can lead to gelation in aqueous solution through locking of the polymer chains into regular structures that act as physical crosslinks. Such molecular assembly is typical for protein- or peptide-based systems [68,154,155], but is not uncommon even with synthetic macromolecules. For example, poly(vinyl alcohol) (PVA) formed physically crosslinked self-healing hydrogels when exposed to repeated freeze-thaw cycles, and the crystallinity as well as the mechanical strength of gels increased with polymer concentration and the number of cycles [73]. PVA gels of 35 wt% showed repeatable rapid healing and evolution of fracture stress: after 10 s recovery, the gels could withstand a stress of around 10 kPa and after 1 h, 40% of initial tensile strength was regained. The crystallites act as physical crosslinks in a hydrogel, while self-healing is favored by concentration-dependent interchain H-bonding and the diffusion of PVA chains over the fractured surface. Hence, self-healing could not occur below a limiting concentration (20 wt%) [73]. The mechanical properties of PVA hydrogels were further enhanced by the addition of H-bonding agent, melamine, that acts as both H-bonding acceptor and donor [156].For example, a gel with 1.5 wt% melamine displayed a tensile strength of 3.11 MPa and 525% elongation at break, while the values for a gel without melamine were 0.74 MPa and 325%, respectively. However, adding melamine reduced the self-healing ability PVA hydrogels (from 86% to 52%) through increasing the gel rigidity, which further led to reduced chain interdiffusion and H-bonding over the fractured surfaces. [157]. Similarly, an interpenetrating hydrogel network of chemically crosslinked PEG and physically crosslinked PVA showed reduced self-healing capacity due to lower PVA chain mobility and lower H-bond forming –OH group density [158].

Examples of proteins and polypeptides that have been shown to form self-healing hydrogels include silk-collagen-like block copolypeptides that showed pH-dependent fibrillar self-assembly [159], diblock copolypeptides composed of charged and hydrophobic segments that exhibited conformation-dependent (α-helix *vs.* β-sheet) gelation and high thermal stability up to 90 °C [160], micelles of elastin-like diblock copolypeptide that self-assembled into rapidly shear-recovering hydrogels upon the addition of Zn^2+^ [161], and bis-maleimide-PEG-linked globular actin (G-actin) that underwent reversible salt-induced self-assembly into fibrillar actin (F-actin) [162]. Pochan, Schneider and coworkers have done extensive work on self-assembling β-hairpin peptides with shear-thinning and self-healing behavior for injectable therapeutic delivery [163]. Recently, engineered peptides containing aggregation-prone and β-sheet-forming C-terminal amyloid β-protein sequences (associated with Alzheimer’s disease) gave rise to amyloid fibril network and weak self-healing hydrogel formation (G′ 40–250 Pa at 10 Hz) [164]. The resulting gels recovered from deformation in 15 min of rest (or in 100 s during step strain experiments) and the recovery mechanism was associated with the stickiness of the hydrophobic surface of amyloid fibrils.

**Figure 14:**
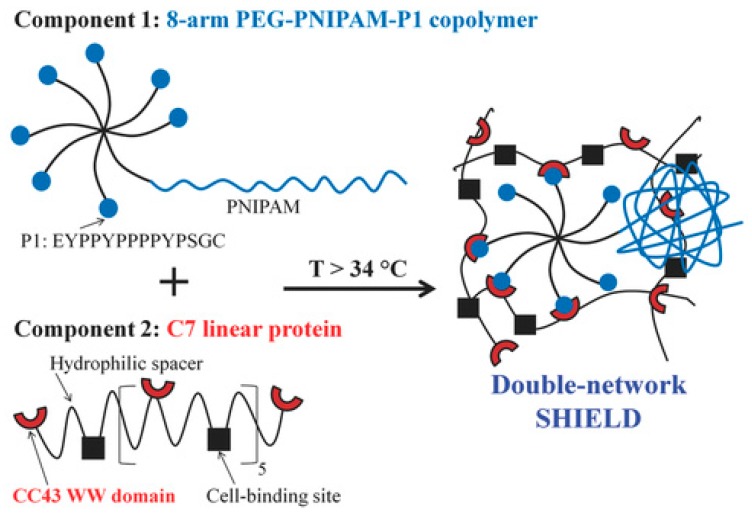
Schematic of a two-component double network “SHIELD”, where **component 1** is an 8-arm PEG with 1 arm conjugated with PNIPAAm and the other 7 arms conjugated with proline-rich peptide (denoted as P1) domains. **Component 2** is a recombinant C7 linear protein copolymer bearing CC43 WW (denoted as C) domains and RGD (arginine-glycine-aspartic acid) cell-binding domains connected by hydrophilic spacers. The formation of double network occurs via peptide recognition and collapse of thermo-responsibe PNIPAAm chains. Reprinted with permission from [165]. Copyright 2016 John Wiley and Sons.

Heilshorn and coworkers [165] prepared an elegant injectable and biocompatible hydrogel that combined synthetic and peptide-based systems and underwent two different physical crosslinking mechanisms. This double network system was based on two components: (1) 8-arm star-like PEG conjugated with one thermo-responsive PNIPAAm chain and seven proline-rich peptide domains (P1); and (2) linear protein copolymer consisting of P1-recognizing units and cell adhesion-favoring RGD arginine-glycine-aspartic acid domains connected by hydrophilic spacers (Figure 14). The first crosslinking occurred *ex vivo* upon mixing the two components, which led to peptide-based recognition and very weak hydrogels with elastic modulus of 13 Pa. The second crosslinking occurred at body temperature upon the collapse of PNIPAAm above its LCST and led to increase in gel strength (G′ ~ 100 Pa). The gels showed reversible rapid self-healing (<2 s) due to reformation of physical network junctions and the process was faster than with gels based on protein-ligand interactions, passive re-entanglement of polymer chains or stepwise reassembly into nanofibers. The gels enhanced stem cell retention at the desired site *in vivo*, reducing the number of injections and cells required for transplantation [165].

## 4. Potential Applications

**Figure 15:**
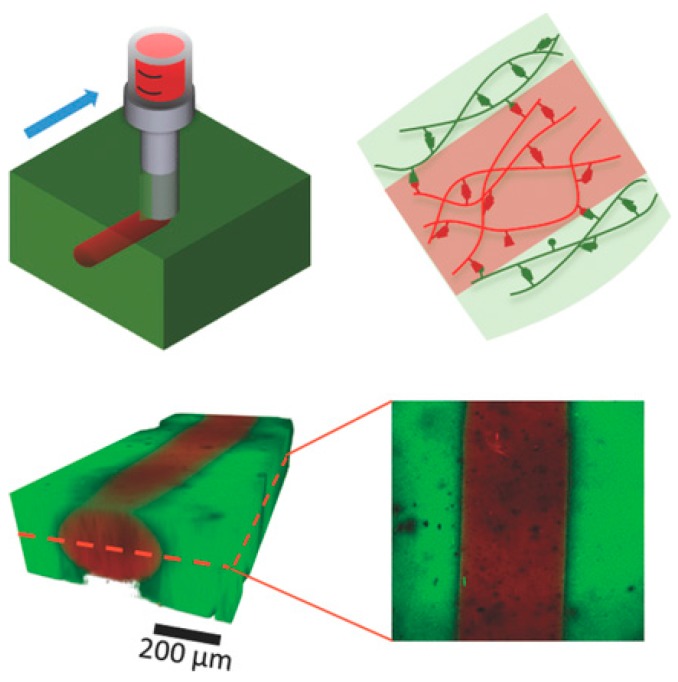
(**Above**): Schematic illustration of 3D printing of a supramolecular gel based on adamantane (Ad, guest) and β-cyclodextrin (β-CD, host) conjugated to hyaluronic acid (HA). Green represents a supramolecular support gel and red the printed ink; (**Below**): 3D reconstruction of a confocal Z-stack of an ink filament (rhodamine-labeled, **red**) printed into a support gel (fluorescein-labeled, **green**). Scalebar: 200 μm. Reprinted from with permission [170]. Copyright 2016 John Wiley and Sons.

Rapid shear thinning or solid-liquid transition and fast structure recovery are key requirements both for injectable hydrogels and hydrogel inks for additive manufacturing. Additive manufacturing technologies, such as 3D printing, enable the fabrication of complex micrometer- and millimeter-scale structures, where hydrogels can be used as “bio-inks” to form constructs that replace, augment or model tissues [166,167,168,169]. The strategies and molecular design criteria of 3D-printable hydrogel inks have been highlighted in a recent review, where non-covalent gelators were categorized into supramolecular polymers, low molecular weight gelators, and macromolecular and colloidal solid particle-based systems [169]. Among these, the naturally occurring, slightly modified biopolymers have so far shown the highest potential for biofabrication owing to their favorable biocompatibility and capability to support cell survival and differentiation in culture. For example, Burdick and coworkers [170] used host-guest complexation-based self-healing gels to construct patterned multicellular structures (Figure 15), where mesenchymal stem cells (MSCs) and 3T3 fibroblasts loaded within a biocompatible network of adamantane- and β-CD-functionalized biopolysaccharide, hyaluronic acid (HA), were successfully printed to their own compartments. Here, a strong 4 wt% HA gel with 40 mol% of β-CD (host) and Ad (guest) units was used as a support gel (G′ ~ 1 kPa at *f* = 1 Hz) and softer 5 wt% shear-thinning gel with 25 mol% of host and guest units was used as an ink (G″ > G′ at *f* = 1 Hz indicating liquid-like behavior; G′ ~300 Pa and G′ > G″ below 1% strain indicating solid-like behavior). Cyclodextrins have widely been used in numerous injectable hydrogels owing to their excellent biocompatibility, weak immunogenicity, and selectivity as supramolecular hosts [44,171]. Secondary covalent crosslinking was introduced via photopolymerization of pendent methacrylate groups (Me-HA) to stabilize the printed gel against chemical or physical perturbations, such as perfusion for the convection of nutrients and removal of cell metabolic waste. As a result, the gels showed increased elastic moduli (G′ >10 kPa) and reduced frequency dependence [170]. Spatially controlled bioprinting of multiple cell types enables the development of organ or tissue constructs that do not require substantial vascularization as well as mini-tissue models for pharmaceutical or cosmetic testing and disease studies [172].

The use of injectable hydrogels provides a minimally invasive strategy for the administration of bioactive molecules and cells in the body [8,173,174]. Simple injection instead of a complicated surgical procedure reduces the infection risks and recovery time in patients and injections can be done to sites that are hard to reach by surgical techniques. Hydrogels can protect protein-based drugs from enzymatic degradation and provide targeted delivery and prolonged release of the drug to the target site [174]. They also provide a specific biological and mechanical three-dimensional environment for cells that guides the regulation of cellular functions during the formation of regenerated tissue. Injection delivery can be done via a syringe or via a catheter and recently, an H-bonded UPy-unit containing hydrogels loaded with a small-molecule drug pirfenidone or a fluorescent model protein mRuby2 were injected into a pig heart via an intramyocardial catheter [175]. The injectable system was a Newtonian fluid with low viscosity at high pH and formed a stiff gel with G′ = 10–20 kPa (at angular frequency ω = 0.1–100 rad/s) after neutralization. Here the liquid-like behavior was necessary for the delivery via a catheter. The gradual degradation of the supramolecular gel and release of the model protein shows promise of these self-healing gels as delivery systems for growth factors and exosomes. In cellular therapy, rapid self-healing of the hydrogel localizes the cells to the targeted area and increases the probability of cell retention after transplantation [165]. The above-mentioned supramolecular HA/β-CD/Ad hydrogel provided improved delivery and retention of endothelial progenitor cells (EPCs) *in vivo* in ischemic myocardium, aiming at the revascularization of myocardium after heart failure. Significant increase in the vascularization, improvement of ventricular function, and reduction of scar formation were observed [176]. Here, both examples of therapies target at the regeneration of ischemic myocardium after infarction.

**Figure 16:**
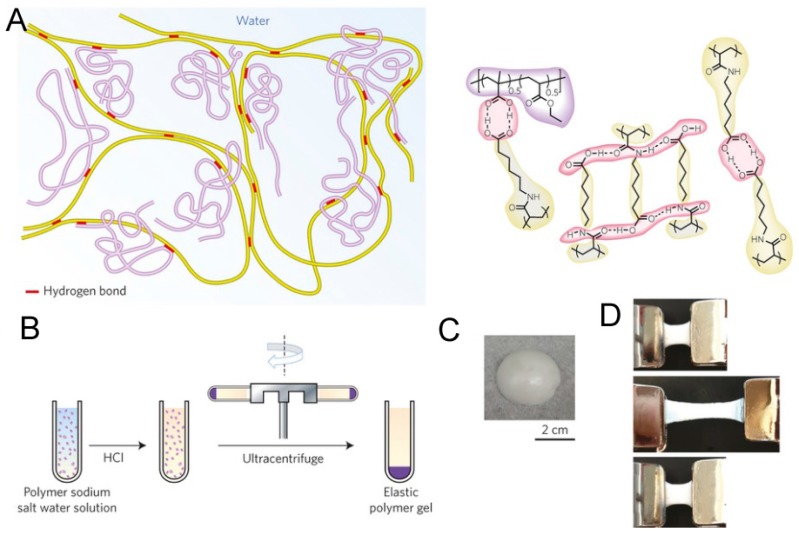
(**A**) Proposed supramolecular polymer gel network. Structures in yellow, synthesized poly(acryloyl 6-aminocaproic acid) (PA6ACA); structures in purple, linear poly(methacrylic acid-co-ethyl acrylate) (EUDRAGIT L 100-55); **red** part, inter-polymer hydrogen bonds; (**B**) Manufacturing process of the polymer gel. (**Left**), the homogeneous solution of PA6ACA sodium salt solution and L 100-55 sodium salt solution with varying polymer weight ratios. (**Middle**), the addition of HCl solution resulting in precipitation; (**Right**), formation of the elastic polymer gel after ultracentrifugation; (**C**) Photo of a piece of the polymer gel obtained after ultracentrifugation; and (**D**) Images of stretch and recovery testing of a polymer gel with PA6ACA:L 100-55 = 1:2. (**Top**), 1.5 cm piece of polymer gel held between two clamps. (**Middle**), stretching of the polymer gel to three times its initial length; (**Bottom**), recovery of the polymer gel 5 min after the external force was removed. Scale bar is 2 cm for (**C**) and (**D**). Reprinted with permission from [180]. Copyright 2016 Nature Publishing Group.

One potential application of self-healing hydrogels is the coating of medical implants. Biocompatible hydrogel coatings with good structural stability can be used to protect orthopedic implants or implanted sensors from biofouling [21,177,178] or to deposit growth factors, cells or peptides on the implant surface [178,179]. The limitation in many bio-applications is the gel strength, which can be enhanced by secondary crosslinking or mixing with other polymers. The latter approach has been used in the preparation of an enteric elastomer (EE, Figure 16) from a mixture of H-bonding PA6ACA polymer, which is structurally similar to traditional enteric polymers, and poly(methacrylic acid-*co*-ethyl acrylate) (EUDRAGIT L 100-55) [180]. The stiffness of the resulting elastomer could be tuned by the ratio of these two polymers and the strongest gel reached the tensile strength of ~3.3 MPa and >900% elongation at break. The elastomer was used to construct a gastric-retentive device with alternating pieces of harder polycaprolactone (PCL) linked together by EE gels. *In vivo* evaluation of the gastric device demonstrated that the device was intact in the acidic conditions of stomach, but dissociated into smaller pieces upon passing to small and large intestine with neutral pH, where H-bonds start breaking upon the deprotonation of acid groups [180]. Hence, these supramolecular materials and their elastomers have strong potential in new gastric-resistant devices for weight control, ingestible electronics, and prolonged drug delivery. Recently, Gong and coworkers demonstrated the reprocessability of tough polyelectrolyte complex-based self-healing hydrogels into capsules, films, and fibers at ambient temperature [181].

## 5. Future Prospects and Conclusions

Self-healing of hydrogels is relatively easier to achieve than in harder materials due to the higher mobility of the chains and the dynamic bonding interactions of the functional groups. Although supramolecular materials have been under extensive research for decades, the focus has only recently shifted on investigating how the reversibility of their physical interactions can be exploited at macroscopic scale for instance, in medical devices or delivery systems. The rapid structure recovery physical gels arising from the diffusion of polymer chains and interacting sites to the damaged area is beneficial in injection- and extrusion-based applications, but achieving a balance between the mechanical properties and recovery rate is challenging. The experimental data of mechanical strength and recovery of the various hydrogels reported are rather scattered and it is difficult to compare them directly due to the differences in experimental conditions and characterization methods used. It is thus difficult to indicate a clear trend that would guide in the selection of the most suitable preparation strategies. Often the choice of the preparation methods depends on the final use of the hydrogels in addition to the required physical properties. Among the physical interactions, hydrogen bonding seems to provide hydrogels of higher strengths with tensile and compression strengths of several MPa. Various techniques to reinforce the hydrogels are highlighted in this review and the most efficient ones combine several crosslinking mechanisms or involve mixing the supramolecularly crosslinking polymer with nano-fibers, -sheets or -particles, or blending with other polymers for better processability and enhanced lifetime.

These approaches show the highest potential for the use of self-healing hydrogels in therapeutic systems and medical devices and are expected to gain significant research interest in the future. Also, incorporating biologically active compounds, such as bile acids or peptides, as supramolecular building blocks is of increasing interest. The presence of pharmacologically active agents or cells may alter the crosslinking and degradation mechanisms of hydrogels, leading to changes in gel deformation and structure recovery and therefore, the long-term stability of each new hydrogel system would need to be investigated under physiological or cell culture conditions for biomedical applications. Physical hydrogels often exhibit time-dependent deformation, referred to as “creep”, which is associated with high dissipative loss modulus in relation to elastic modulus and may affect the stem cell fate choice and tissue development in cell-seeded hydrogels [182]. In addition, the supramolecular carrier system should not interfere with the biological activity of the therapeutic agent. Self-healing physical gels provide a promising platform for building dynamic devices and systems, especially upon the recent innovations in 3D printing, and combining supramolecular and biotechnological approaches shows a great promise for the development of structurally controlled materials with tailored mechanical properties.

## Abbreviations

The following abbreviations are used in this manuscript:

Ad	adamantane
AdCANa	sodium adamantanecarboxylate
AAm	acrylamide
AAZ	carboxybetaine acrylate
Alg	alginate
ASAP	sodium polyacrylate
CB[n]	cucurbit[n]uril, *n* = number of glycouril units
CBMAA-3	(3-methacryloylaminopropyl)-(2-carboxyethyl)dimethylammonium-(carboxybetaine methacrylamide)
CD	cyclodextrin
Chol	cholesterol
CNS	clay nanosheets
DMAEMA	2-(dimethylamino)ethyl methacrylate
DMAPS	3-dimethyl(methacryloyloxyethyl) ammonium propane sulfonate
CTAB	cetyl trimethyl ammonium bromide
DMA	*N*,*N*′-dimethylacrylamide
DODAB	dimethyldioctadecylammonium bromide
EE	enteric elastomer
EPC	endothelial progenitor cell
Fc	ferrocene
G′	storage modulus
G″	loss modulus
GO	graphene oxide
HA	hyaluronic acid
HEC	hydroxyethyl cellulose
HEMA	2-hydroxyethyl methacrylate
MEO2MA	2-(2-methoxyethoxy)ethyl methacrylate
MSC	mesenchymal stem cell
MV	methylviologen
NFC	nanofibrillar cellulose
NIPAAm	*N*-isopropylacrylamide
Np	naphthyl
PA6ACA	poly(6-acryloyl-6-aminocaproic acid)
PAAc	poly(acrylic acid)
PEG	poly(ethylene glycol)
PLGA	poly(L-glutamic acid)
PPG	poly(propylene glycol)
PVA	poly(vinyl alcohol)
RGD	arginine-glycine-aspartic acid
SCMHBMA	2-(3-(6-methyl-4-oxo-1,4-dihydropyrimidin-2-yl)ureido)ethyl methacrylate
SDS	sodium dodecyl sulfate
SEM	scanning electron microscopy
STMV	methyl viologen-functionalized cationic polystyrene
UPy	2-ureido-4[1*H*]pyrimidinone
